# A nomogram for predicting survival in patients with advanced (stage III/IV) pancreatic body tail cancer: a SEER-based study

**DOI:** 10.1186/s12876-022-02362-2

**Published:** 2022-06-03

**Authors:** Huaqing Shi, Zhou Chen, Shi Dong, Ru He, Yan Du, Zishun Qin, Wence Zhou

**Affiliations:** 1 The First Clinical Medical College, Lanzhou University, Lanzhou, China; 2 Department of General Surgery, Lanzhou University Second Hospital, Lanzhou, Gansu 730000 China; 3 School of Stomatology, Lanzhou University, Lanzhou, China

**Keywords:** Nomogram, Pancreatic body tail cancer, Prognosis, Overall survival, SEER database

## Abstract

**Objective:**

Pancreatic body tail carcinoma (PBTC) is a relatively few pancreatic cancer in clinical practice, and its specific clinicopathological features and prognosis have not been fully described. In this study, we aimed to create a nomogram to predict the overall survival (OS) of patients with advanced PBTC.

**Methods:**

We extracted clinical and related prognostic data of advanced PBTC patients from 2000 to 2018 from the Surveillance, Epidemiology, and End Results database. Independent prognostic factors were selected using univariate and multivariate Cox analyses, and a nomogram was constructed using R software. The C-index, area under the curve (AUC) of receiver operating characteristic curves, calibration curves, and decision curve analysis (DCA) were used to assess the clinical utility of the nomogram. Finally, OS was assessed using the Kaplan–Meier method.

**Results:**

A total of 1256 patients with advanced PBTC were eventually included in this study. Age, grade, N stage, M stage, surgery, and chemotherapy were identified as independent risk factors using univariate and multivariate Cox regression analyses (*p* < 0.05). In the training cohort, the calibration index of the nomogram was 0.709, while the AUC values of the nomogram, age, grade, N stage, M stage, surgery, and chemotherapy were 0.777, 0.562, 0.621, 0.5, 0.576, 0.632, and 0.323, respectively. Meanwhile, in the validation cohort, the AUC values of the nomogram, age, grade, N stage, M stage, surgery, and chemotherapy were 0.772, 0.551, 0.629, 0.534, 0.577, 0.606, and 0.639, respectively. Good agreement of the model in the training and validation cohorts was demonstrated in the calibration and DCA curves. Univariate survival analysis showed a statistically significant effect of age, grade, M stage, and surgery on prognosis (*p* < 0.05).

**Conclusion:**

Age, grade, M stage, and surgery were independently associated with OS, and the established nomogram was a visual tool to effectively predict OS in advanced PBTC patients.

## Introduction

Pancreatic cancer is a highly malignant solid tumour of the digestive organs with a poor prognosis, of which pancreatic adenocarcinoma (PAAD) of the pancreatic duct accounts for more than 90% of pancreatic cancers [1]. Approximately 80% of PAAD patients have locally advanced or distant metastases at the time of presentation and are lost to surgery [2]. Pancreatic cancer is the fourth leading cause of cancer death [3], with a 5-year survival rate of less than 5% [3], of which only 15–20% of patients with PAAD are diagnosed at the surgically resectable stage [4], and surgical resection is currently the only possible curative treatment; however, even after resection, the 5-year survival rate is less than 20% due to the high frequency of distant metastases and local recurrence [5]. The pancreas head is near the duodenum, the pancreas body tail is located behind the stomach, and the pancreas tail extends towards the splenic hilum. Islet cells are mainly distributed in the tail of the body [6]. Clinically, PHC is mostly derived from pancreatic duct epithelial cells, while PBTC is mostly derived from acinar and islet cells. Intraductal papillary mucinous neoplasms (IPMNs) and mucinous cystic neoplasms (MCNs) have been definitively indicated as precursors of pancreatic ductal adenocarcinoma [7, 8]. A study found that MCNs (as defined by ovarian-type stroma) mainly occur in female patients (> 95%) and are predominantly located in the body-tail region of the pancreas (> 95%) [9]. Milanetto et al. found that immunohistochemical analysis showed positivity for CK7 expression in 100% of MCNs. For operable patients, PBTC mainly performs standard pancreatic resections (distal pancreatectomy and pancreaticoduodenectomy) [10].

PBTC is relatively uncommon in clinical practice. Approximately 60–70% of PAADs are located in the head of the pancreas, and the rest, with a similar rate of approximately 15%, are located in the body and tail of the pancreas, respectively [11]. However, there are fewer cases of PBTC in clinical practice, and the prognosis and suitability of the choice of treatment remain unclear. Previous studies have shown that the prognosis of PBTC in PAAD is poor [12]. Currently, surgery is the only curable option, and radiotherapy combined with gemcitabine chemotherapy remains the main treatment for patients with advanced disease [13–15].

In the present study, we defined patients with stage III to IV PBTC as having advanced PBTC according to the AJCC-TNM 7th edition. The nomogram is a new multivariate model capable of integrating the relative contribution of each prognostic variable to the prognostic prediction outcome [16, 17]. Although the role of the nomogram has been validated in various cancers [18–20], its use in patients with advanced PBTC applications has not been adequately studied. Therefore, we retrospectively analysed 1256 advanced PBTC patients from the SEER database to establish a valuable nomogram based on a Cox proportional hazards regression model to predict 1-, 3-, and 5-year survival in advanced PBTC patients and to validate it.

## Materials and methods

### Data extraction

Our study used the SEER database, which is one of the more widely used and reliable publicly-available cancer databases, covering approximately 28% of the US population [21]. Clinicopathological data on advanced PBTC were collected from the SEER database (Database name = Incidence—SEER Research Plus Data, 18 Registries, Nov 2020 Sub (2000–2018)—Linked To County Attributes—Total U.S., 1969–2019 Counties) using SEER*Stat 8.3.9.2 (http://seer.cancer.gov).

We searched the SEER database for patients with a tumour location of pancreatic body tail and a primary tumour of pancreatic body tail cancer with positive histological pathology confirmation. Well-established data on age, race, tumour stage and treatment types were available, and complete and valid follow-up records were available with no missing follow-up data. The study protocol was conducted in accordance with the Declaration of Helsinki (as revised in 2013). Since this is a retrospective study and the patient information in the SEER database is anonymous, ethical consent is not required for using these data [22, 23].

### Characteristic variables and survival data

Patient clinicopathological data were obtained from the following datasets: age, sex, race, TNM and histological staging, whether surgery was performed, whether radiotherapy was administered, survival data, and vital status. TNM stage was manually adjusted according to the American Joint Committee on Cancer (AJCC) seventh edition criteria. To construct a Cox regression-based analysis, patients were grouped by age at diagnosis, with 65 years of age being the cut-off between groups. We included those in the radiotherapy and chemotherapy group who were unknown about radiotherapy and chemotherapy in the no radiotherapy and chemotherapy group. The primary endpoint of this analysis was OS derived from vital status and months of survival, with months of survival calculated from the day of surgery to the last follow-up or death. Patients who survived less than 1 month were coded as having zero survival time in the SEER database, and the sample with zero survival time was excluded from this study.

### Statistical analysis

We used the χ2 test (or Fisher’s exact test) for the clinicopathological characteristics of the study population. Survival analysis was performed using Kaplan–Meier estimation and the log-rank test. For the construction of Cox proportional hazards regression models, they were first calculated using R 4.1.2 (http://www.r-project. org/) and validated using SPSS software (IBM corporation, version 22.0.0) calculations, including univariate analysis (UVA) and multivariate analysis (MVA), and hazard ratios (HRs) and 95% confidence intervals (95% CIs) were calculated to assess the impact of clinical indicators on patient prognosis. A nomogram was constructed based on the MVA results. Independent prognostic factors were used to draw nomograms to predict 1-, 3-, and 5-year OS in patients with advanced PBTC. In this study, we used the training cohort to construct nomograms that were validated in the validation cohort. AUCs of ROC curves and C-indices were calculated, and calibration curves were plotted to assess the predictive power of the model. DCA was used to assess the utility of nomograms for decision-making [24]. Kaplan–Meier survival curves were plotted according to age, grade, N stage, M stage, surgery, and chemotherapy. Analyses were performed with R using the rms, Hmisc, lattice, survival, formula, ggplot2, rmda, survminer, pROC, timeROC, and foreign packages. *p* values < 0.05 were considered statistically significant.

## Results

### Patient clinicopathological and demographic characteristics

We performed a rigorous selection process that resulted in 1256 patients with advanced PBTC from the SEER database search. According to a 7:3 ratio, 879 patients were randomly assigned to the training cohort, and 377 patients were randomly assigned to the validation cohort using the random sampling method. The distribution of all variables was similar between the two groups and was not statistically significant (*p* > 0.05). Detailed clinicopathological and demographic information is presented in Table 1. In the total sample, patients were divided into two age groups (< 65 and ≥ 65 years), with 55.6% of patients aged ≥ 65 years. Males accounted for 53.3%, the majority were white (n = 955), 42% were poorly differentiated in grade III, and 81% were AJCC stage IV patients. The TNM stage was predominantly T4 (n = 485), 61% in N0, and 81% in M1. For treatment, only 25.3% of patients underwent surgery, 12.7% underwent radiotherapy, and 65.1% underwent chemotherapy.

**Table 1:** The baseline level of 1256 patients

Variables	n (%)	Train cohort n (%)	Validation cohort n (%)	*P*
Age		879	377	
< 65	558 (44.4)	391 (44.5)	167 (44.3)	1.000
≥ 65	698 (55.6)	488 (55.5)	210 (55.7)	
Sex				
Male	670 (53.3)	472 (53.7)	198 (52.5)	0.748
Female	586 (46.7)	407 (46.3)	179 (47.5)	
Race				
White	955 (76.0)	667 (75.9)	288 (76.4)	0.371
Black	210 (16.7)	153 (17.4)	57 (15.1)	
Others	91 (7.2)	59 (6.7)	32 (8.5)	
Grade				
I (Well)	186 (14.8)	118 (13.4)	68 (18.0)	0.092
II (Moderately)	511 (40.7)	373 (42.4)	138 (36.6)	
III (Poorly)	528 (42.0)	365 (41.5)	163 (43.2)	
IV (Undifferentiated)	31 (2.5)	23 (2.6)	8 (2.1)	
AJCC stage				
III	239 (19.0)	166 (18.9)	73 (19.4)	0.905
IV	1017 (81.0)	713 (81.1)	304 (80.6)	
T stage				
T1	32 (2.5)	20 (2.3)	12 (3.2)	0.519
T2	332 (26.4)	227 (25.8)	105 (27.9)	
T3	407 (32.4)	294 (33.4)	113 (30.0)	
T4	485 (38.6)	338 (38.5)	147 (39.0)	
N stage				
N0	766 (61.0)	542 (61.7)	224 (59.4)	0.494
N1	490 (39.0)	337 (38.3)	153 (40.6)	
M stage				
M0	239 (19.0)	166 (18.9)	73 (19.4)	0.905
M1	1017 (81.0)	713 (81.1)	304 (80.6)	
Surgery				
No	938 (74.7)	645 (73.4)	293 (77.7)	0.121
Yes	318 (25.3)	234 (26.6)	84 (22.3)	
Radiotherapy				
No	1096 (87.3)	765 (87.0)	331 (87.8)	0.778
Yes	160 (12.7)	114 (13.0)	46 (12.2)	
Chemotherapy				
No	438 (34.9)	304 (34.6)	134 (35.5)	0.793
Yes	818 (65.1)	575 (65.4)	243 (64.5)	

*AJCC* American joint committee on cancer

### Identification of prognostic factors

Univariate analysis showed that seven factors with a *p* value < 0.05 were closely associated with patient OS. Considering the prognostic role of N stage and chemotherapy on patients, we included them in multivariate analysis. Due to a potential correlation with TNM staging, AJCC staging was excluded from MVA to avoid covariance between factors. The results of the UVA and MVA Cox regression analysis models are shown in Table 2. Age (*P* < 0.05), grade (*P* < 0.05), N stage (*P* < 0.05), M stage (*P* = 0.033), surgery (*P* < 0.05), and chemotherapy (*P* < 0.05) were independent predictors of survival.

**Table 2:** Univariate and multivariate cox regression model of overall survival for advanced PBTC

Variables	Univariate analysis			Multivariate analysis		
	HR	95% CI	*P*	HR	95% CI	*P*
Age						
< 65	Ref					
≥ 65	1.491	1.324–1.678	0.000	1.355	1.202–1.528	0.000
Sex						
Male	Ref					
Female	0.945	0.841–1.061	0.339			
Race						
White	Ref					
Black	1.041	0.89–1.218	0.612			
Others	0.987	0.783–1.244	0.912			
Grade						
I (Well)	Ref					
II (Moderately)	2.328	1.913–2.832	0.000	2.280	1.863–2.790	0.000
III (Poorly)	3.159	2.597–3.844	0.000	2.854	2.326–3.502	0.000
IV (Undifferentiated)	2.379	1.589–3.561	0.000	2.419	1.610–3.636	0.000
AJCC stage						
III	Ref					
IV	1.306	1.126–1.514	0.000			
T stage						
T1	Ref					
T2	1.755	1.175–2.620	0.006	1.029	0.687–1.540	0.891
T3	1.246	0.837–1.856	0.279	0.941	0.629–1.408	0.769
T4	1.393	0.937–2.069	0.101	0.955	0.633–1.442	0.827
N stage						
N0	Ref					
N1	0.953	0.846–1.073	0.424	1.246	1.101–1.409	0.000
M stage						
M0	Ref					
M1	1.306	1.126–1.514	0.000	1.233	1.017–1.494	0.033
Surgery						
No	Ref					
Yes	0.337	0.290–0.391	0.000	0.350	0.297–0.413	0.000
Radiotherapy						
No	Ref					
Yes	0.735	0.618–0.873	0.000	0.891	0.740–1.072	0.221
Chemotherapy						
No	Ref					
Yes	0.972	0.858–1.102	0.662	0.699	0.612–0.798	0.000

*PBTC* pancreatic body tail cancer, *HR* hazard ratio, *CI* confidence intervals *AJCC* American joint committee on cancer

### Construction of the prognostic nomogram

**Fig. 1:**
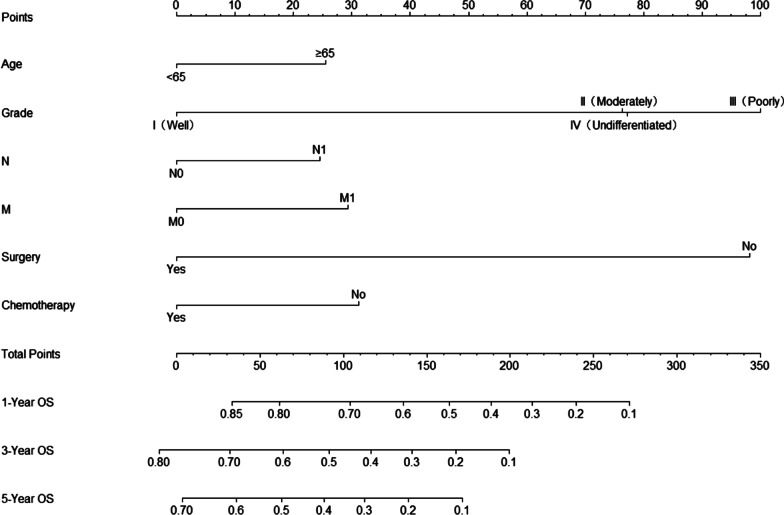
Nomogram for predicting 1-, 3-, and 5-year OS rates in patients with advanced PBTC. The points for each variable can be estimated by plotting a vertical line upwards from the patient's variable values to the top axis marked as “points”. A vertical line is drawn downwards from the sum of all variable values on the "total points" axis to calculate 1-, 3-, and 5-year OS rates. OS: overall survival; PBTC: pancreatic body tail cancer

We constructed a nomogram for OS based on independent prognostic factors selected using multivariate Cox analysis (Fig. 1). The nomograms showed that grade contributed the most to predicting OS in patients with advanced PBTC, followed by surgery, chemotherapy, M-stage, age, and N-stage. Each significant variable was assigned a weighted score ranging from 1 to 100. These scores were then summed to determine the value of the vertical intersection of the probability of survival axis and the total score axis, which implies the prognosis for survival at 1, 3, and 5 years for patients with advanced PBTC.

### Nomogram validation

**Fig. 2:**
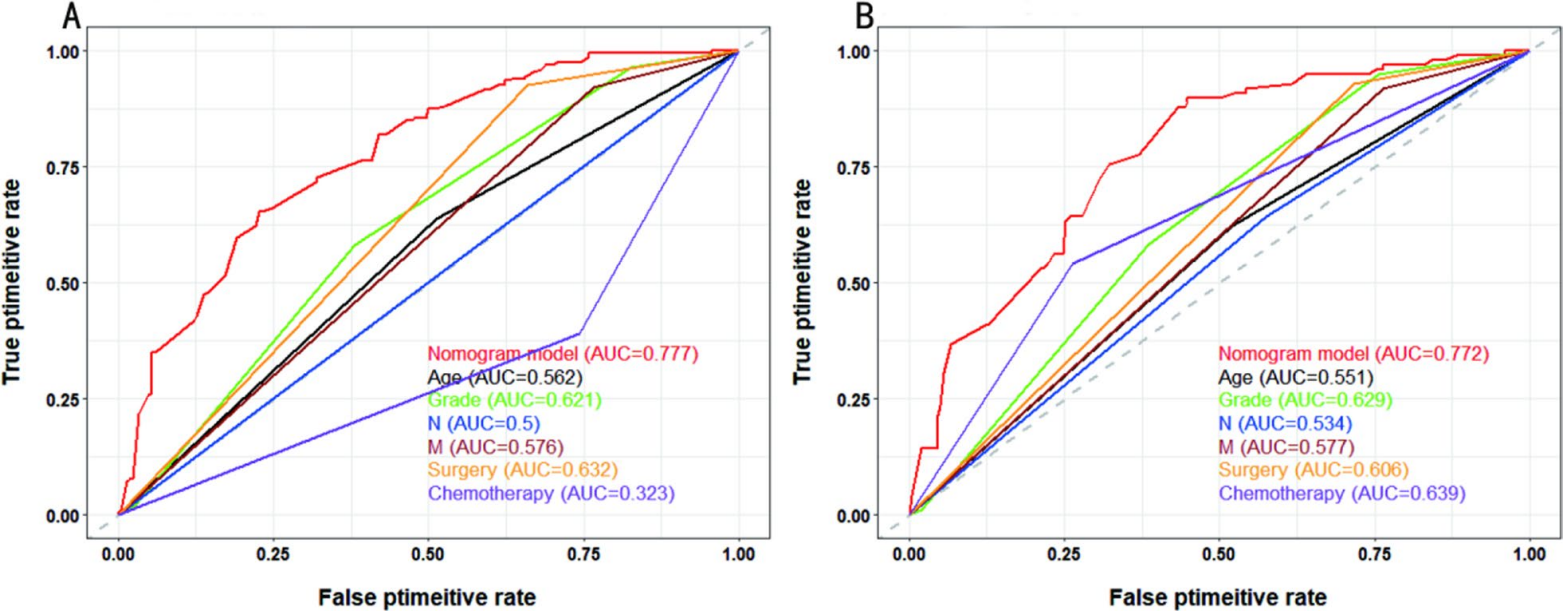
Correlation ROC curve analysis of prognostic models for advanced PBTC. Different colours represent different curves, where red, black, green, blue, brown, orange, and purple represent the nomogram model, age, grade, N, M, surgery, and chemotherapy, respectively. **A** In the training cohort, the AUC values for the nomogram, age, grade, N stage, M stage, surgery and chemotherapy were 0.777, 0.562, 0.621, 0.5, 0.576, 0.632 and 0.323, respectively. **B** In the validation cohort, the AUC values for the nomogram, age, grade, N stage, M stage, surgery and chemotherapy were 0.772, 0.551, 0.629, 0.534, 0.577, 0.606, and 0.639, respectively. ROC: receiver operating characteristic; AUC: area under the curve; PBTC: pancreatic body tail cancer

**Fig. 3:**
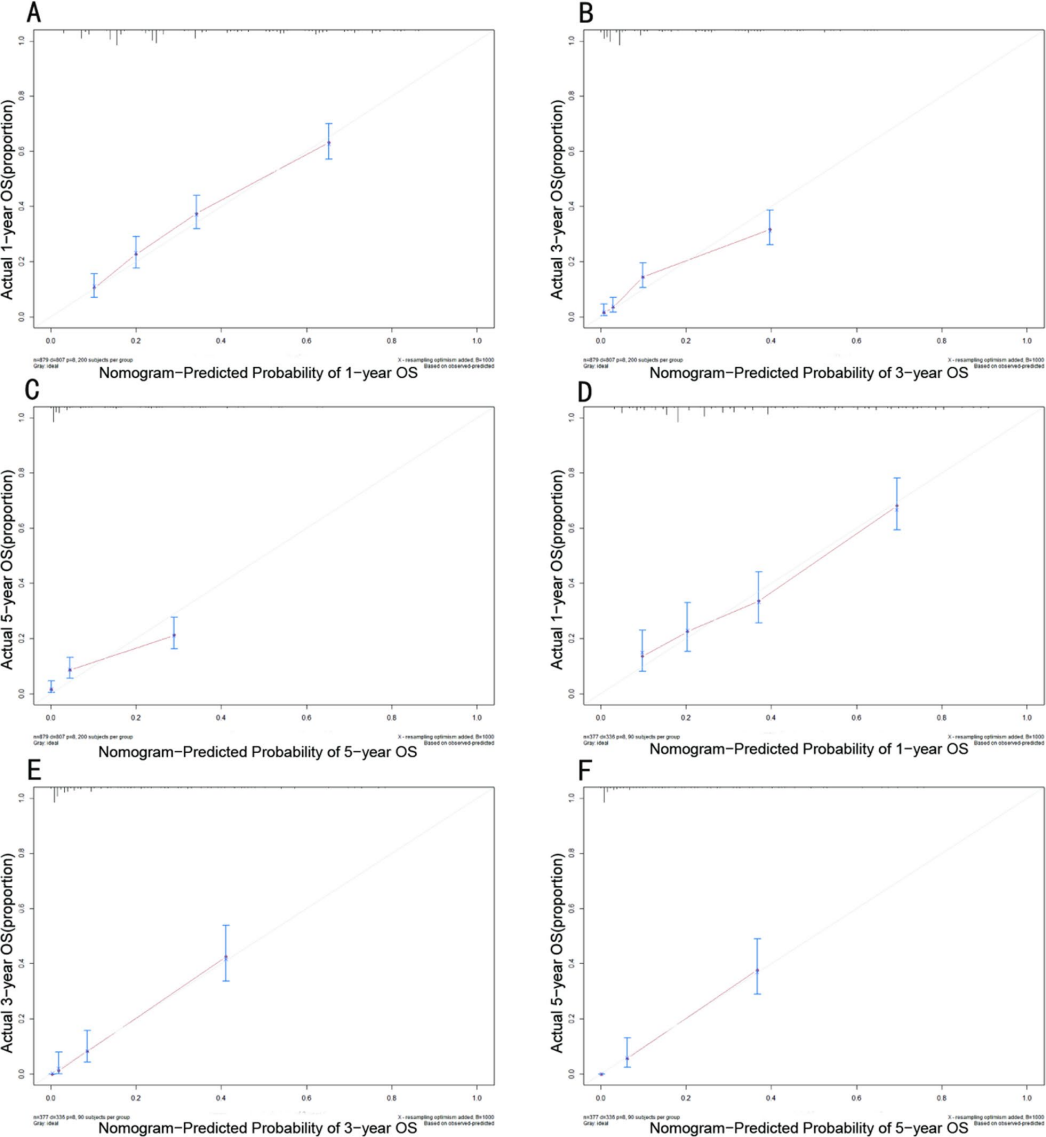
Calibration curves in the training cohort and validation cohort. **A**–**C** Nomogram calibration curves for predicting OS in advanced PBTC patients at 1, 3, and 5 years in the training cohort. **D**–**F** Nomogram calibration curves predicting the OS for advanced PBTC patients at years 1, 3, and 5 in the validation cohort. The red line represents an equal probability of observed and predicted values. OS: overall survival; PBTC: pancreatic body tail cancer.

In our study, the C-index, ROC curve, and calibration plots were used to assess the utility of the nomogram. In the training cohort, the C-index value was 0.709, and the AUC value of the ROC curve was 0.777 (Fig. 2A), while age, grade, N stage, M stage, surgery, and chemotherapy were 0.562, 0.621, 0.5, 0.576, 0.632 and 0.323, respectively. Meanwhile, the calibration plots of the OS nomograms showed high agreement between the nomogram predictions and actual data (Fig. 3A–C). In the validation group, the C-index value of the nomogram for predicting OS was 0.708. As shown in Fig. 2B, the AUC value was 0.772, while age, grade, N stage, M stage, surgery and chemotherapy were 0.551, 0.629, 0.534, 0.577, 0.606, and 0.639, respectively. In addition, calibration plots in the validation cohort showed satisfactory performance of the predicted and actual values (Fig. 3D–F).

### Comparison of the nomogram and AJCC TNM staging systems

**Fig. 4:**
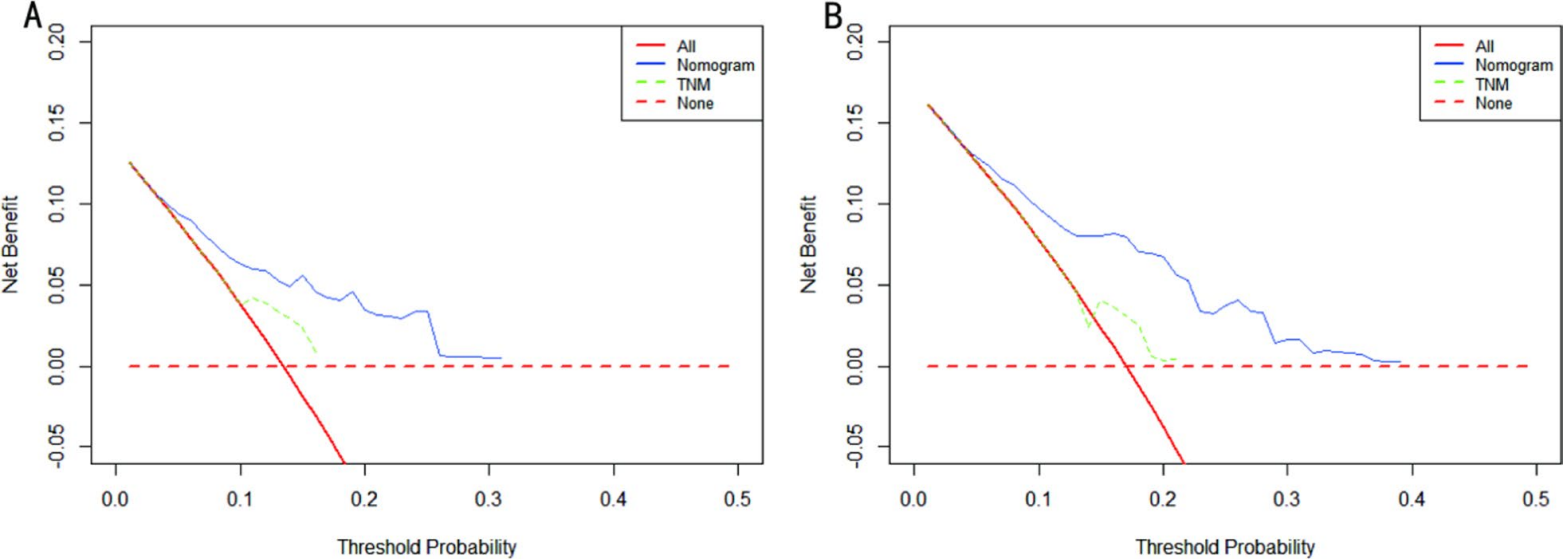
DCA curves of the nomogram and TNM staging system. **A** DCA curves for OS in the training cohort; **B** DCA curves for OS in the validation cohort. The y-axis represents the net benefit, and the x-axis represents the corresponding risk threshold. The solid red line represents all patients who died during the follow-up period. The red dashed line represents no patient deaths during the follow-up period. The solid blue line represents the net benefit of the nomogram at different risk thresholds. The green dashed line represents the net benefit of TNM staging at different risk thresholds. DCA: decision curve analysis; OS: overall survival.

We compared the nomogram model with the seventh edition of the AJCC TNM staging. DCA was used to assess the utility of the new model for predicting prognosis. As shown in Fig. 4, this new model is clinically useful and has a greater net gain in predicting OS than the AJCC staging system in both the training and validation cohorts.

**Fig. 5:**
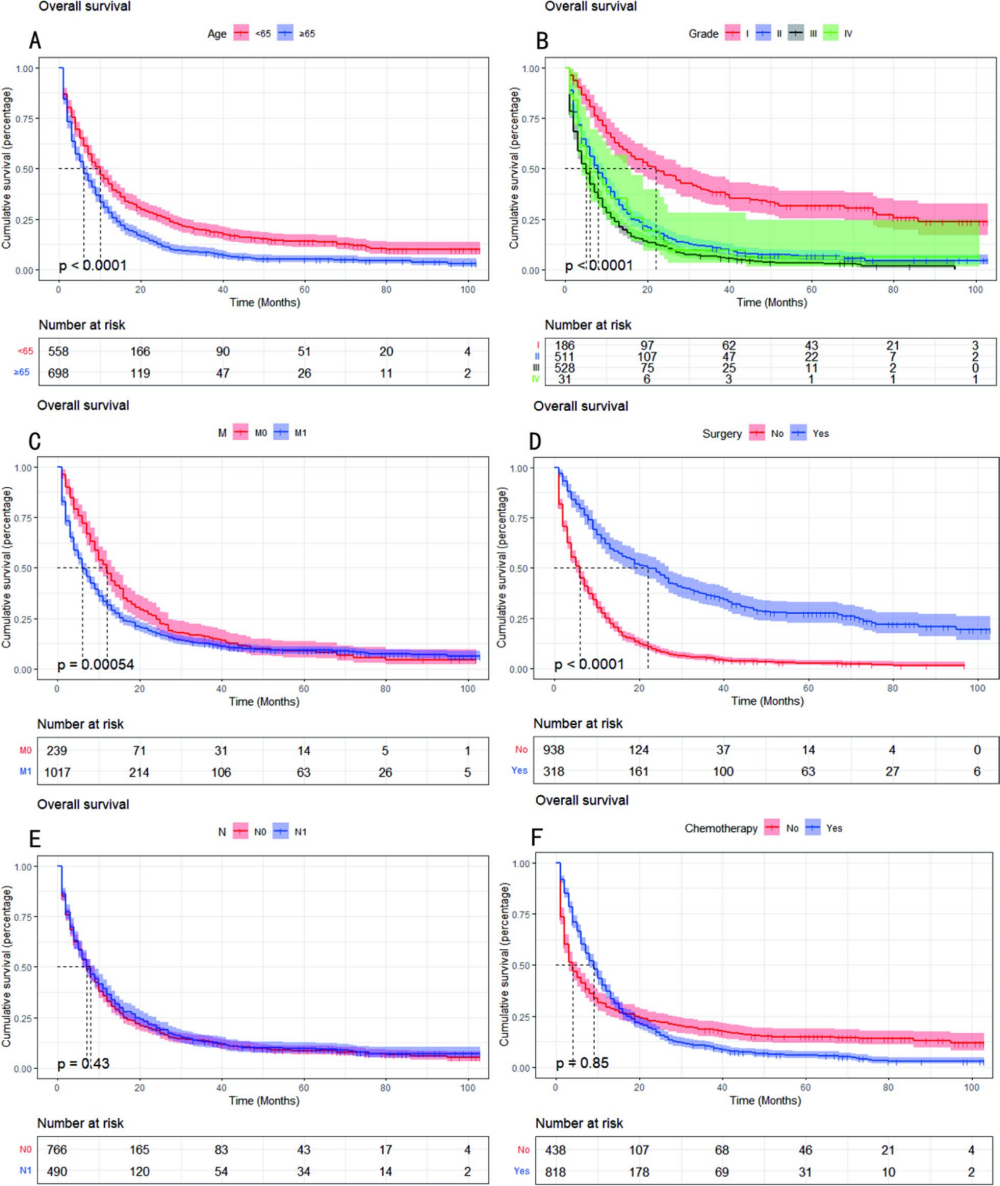
Kaplan–Meier curves show overall survival in patients with advanced PBTC. **A** Kaplan–Meier curves for OS in age groups. **B** Kaplan–Meier curves for OS in the grade groups. **C** Kaplan–Meier curves for OS in the M groups. **D** Kaplan–Meier curves for OS in the surgery groups. **E** Kaplan–Meier curves for OS in the N groups. **F** Kaplan–Meier curves for OS in the chemotherapy groups. *P* < 0.05 was considered statistically significant. OS: overall survival; PBTC: pancreatic body tail cancer

In addition, the cumulative survival of patients in each group is shown in Fig. 5. There was a significant difference in the likelihood of survival between the groups for patients aged < 65 and ≥ 65 years (*P* < 0.05, Fig. 5A), which showed that patients aged ≥ 65 years had a significantly worse OS. Among the grade-stage patients, grade I had the best prognosis, and among the M-stage patients, M0 had a better prognosis than M1 (*P* < 0.05, Fig. 5B, C). The prognosis was significantly better for patients who underwent surgery than those who did not (*P* < 0.05, Fig. 5D). However, the effect of N stage and chemotherapy on OS was not significant (*P* > 0.05, Fig. 5E, F).

## Discussion

Pancreatic cancer is highly malignant and is currently the seventh leading cause of cancer death worldwide, ranking fourth in Europe and the United States after lung, colorectal, and breast cancer, and is expected to become the third by 2030 [25], while its publication rate is also on the rise in China [26]. Because PBTC is uncommon in pancreatic cancer, and there are few studies on advanced PBTC, its incidence increases with the total number of patients with pancreatic cancer, so we need to further study it to guide clinical treatment. Currently, the TNM staging system is usually used to predict and evaluate the prognosis of PBTC patients in clinical practice, but it has limitations because it does not consider other factors affecting survival. Therefore, we constructed a nomogram model for advanced PBTC patients to comprehensively and accurately predict survival prognosis to fill the gap in applying this model in advanced PBTC.

In this study, we analysed the clinical information related to 1256 patients diagnosed with advanced PBTC, established a prognostic model, and validated it. We found that there were more nonsurgical patients, patients undergoing radiotherapy and chemotherapy, and a higher proportion of patients with M1 stage and high T stage, but in grade stage, we found that advanced PBTC patients were predominantly intermediate and poorly differentiated, while undifferentiated accounted for the least (2.5%). Additionally, the analysis of the N stage revealed that N0 accounted for a larger proportion, while the proportion of patients with lymphatic metastases was only 39%. MORITA K et al. found that a reduction in lymphatic infiltrative metastasis was associated with adjuvant therapy such as radiotherapy and chemotherapy [27]; the exact mechanism of which has not been elucidated and may be related to the destruction of small lymphatic vessels associated with cancer and the killing of tumour cells. In addition, several studies have shown that the number of lymph node metastases and positive nodes are strongly associated with overall survival in patients with resectable pancreatic ductal adenocarcinoma [28, 29]. However, the number of regional lymph nodes retrieved and evaluated is influenced by the anatomical site of lymph nodes, the extent of debridement, and the accuracy of pathological examination [30].

Diagnostically, the overall sensitivity of endoscopic ultrasound-guided fine-needle aspiration (EUS-FNA) for the diagnosis of pancreatic cancer is approximately 90% due to its high accuracy and low complications [31]. In clinical practice, EUS-FNA has become the most accurate method for the preoperative diagnosis of pancreatic malignancies [32]. However, a routine preoperative biopsy is generally not recommended to prevent tumour spread from needle implantation. For patients who are difficult to identify and require radiotherapy or chemotherapy, the pathological examination can be performed using a needle biopsy. For patients with PBTC without distant metastases, radical resection is the primary approach, with pancreatic body and tail resection to preserve pancreatic function. Laparoscopic surgery may be used more often in clinical practice, and some studies have found that it may have better advantages in reducing complications such as bleeding [33]. This is because excessive blood loss can cause tumour cell spread and elevated levels of interleukins 1 and 6, leading to early tumour recurrence and poorer survival rates [34, 35]. Inoperable pancreatic cancer has traditionally been treated with gemcitabine, but it is easily resistant and has poor survival rates. Failure of clinical treatment in patients with pancreatic cancer is often due to the heterogeneity of the disease [36]. In contrast, combination chemotherapy with FOLFORINOX (a combination of folinic acid, 5-fluorouracil (5-FU), irinotecan, and oxaliplatin, or a combination of gemcitabine and nab-paclitaxel) and gemcitabine is more effective than gemcitabine alone. Albumin-bound paclitaxel (nab-paclitaxel) was approved by the Food and Drug Administration (FDA) in 2013 for the treatment of pancreatic cancer in combination with gemcitabine [37]. However, in most patients with advanced disease, these treatments only prolong survival by a few months, and the combination therapy also leads to a significant increase in toxicity [38]. Targeted drugs for pancreatic cancer are also in development and exploration [36]. We identified age, grade, M stage, and surgery as independent prognostic factors based on Cox regression and Kaplan- Meier survival analyses. In advanced PBTC, Kaplan–Meier survival analysis of N stage and chemotherapy was not statistically significant.

In our study, the nomogram was constructed based on multivariate outcomes, which need to be validated to avoid overfitting and improve generalizability [39]. The C-index and AUC values were used to assess the accuracy and discrimination of the nomogram for OS in patients with advanced PBTC [40]. Here, the C-index was 0.709 and 0.708 in the training and validation cohorts, respectively, and the AUC values were 0.777 and 0.772. Calibration curves were plotted to demonstrate the good performance of this novel model [16]. In addition, DCA was used to ensure that the nomogram was a relatively good predictor of survival time in patients with advanced PBTC.

Our study also has some limitations. First, it is a retrospective study based on the SEER database. There may be some degree of bias because the information recorded in the SEER database is incomplete. Second, information on possible important prognostic factors was incompletely recorded, such as the absence of detailed chemotherapy regimens and doses. Third, this study is a single retrospective analysis, and more prospective and multicentre studies are needed to validate the model; thus, the model will be more convincing. Finally, since the sample size for advanced PBTC is relatively small, further studies with larger sample sizes are required.

## Conclusion

We constructed prediction models based on the SEER database to predict advanced PBTC, and their reliability and applicability were validated. Age, grade, M-stage, and surgery were independently associated with OS. The nomogram can be used to effectively predict 1-year, 3-year, and 5-year OS in these patients.

## Data Availability

The data used and analysed during this study are available in an open database, the Surveillance, Epidemiology, and End Results (SEER) 18 Registries Data (https://seer.cancer.gov/).

## Abbreviations

PBTC: Pancreatic body tail cancer
OS: Overall survival
SEER: Surveillance, epidemiology, and end results
AUC: Area under the curve
ROC: Receiver operating characteristic
DCA: Decision curve analysis
PAAD: Pancreatic adenocarcinoma of the duct
AJCC: American joint committee on cancer
UVA: Univariate analysis
MVA: Multivariate analysis
HRs: Hazard ratios
95% CIs: 95% confidence intervals
EUS-FNA: Endoscopic ultrasound-guided fine-needle aspiration
5-FU: 5-fluorouracil
nab-paclitaxel: And albumin-bound paclitaxel
FOLFORINOX: A combination of folinic acid, 5-fluorouracil, irinotecan, and oxaliplatin, or a combination of gemcitabine and nab-paclitaxel
FDA: Food and drug administration
IPMN: Intraductal papillary mucinous neoplasms
MCN: Mucinous cystic neoplasms
